# Editorial: The role of genetic alterations in thyroid carcinoma

**DOI:** 10.3389/fendo.2022.990668

**Published:** 2022-08-23

**Authors:** Joachim Feldkamp

**Affiliations:** Academic Department of Endocrinology and Diabetes, General Internal Medicine, Infectiology, Medical School and University Medical Center East Westphalia-Lippe, Klinikum Bielefeld, Bielefeld University, Bielefeld, Germany

**Keywords:** thyroid carcinoma, differentiated, anaplastic, mutation, medullary thyroid cancer

Thyroid carcinomas are detected with increasing incidence rates in many countries. This is mainly contributed to more frequent diagnostic procedures around the world.

Most of the tumors are papillary and follicular thyroid carcinomas, followed by non-follicular medullary thyroid carcinomas and anaplastic carcinomas. The clinical spectrum shows a wide range from sometimes almost benign disease in patients with papillary microcarcinomas, especially in the elderly and when detected incidentally, to the rare cases of anaplastic thyroid carcinomas and primary squamous cell carcinoma of the thyroid with an unfavorable outcome.

For some types of thyroid carcinomas genetic causes are best known. 25% of medullary thyroid carcinomas (MTC) arise in the context of multiple endocrine neoplasia type 2 A and B. The knowledge of causative genetic alterations on cancer development has led to a strong improvement in the survival of patients with MEN-2 disease. A genotype-phenotype association showing different outcomes with specific mutations has led to individualized therapy strategies with prophylactic surgery in many cases, detected by family screening. Hansen et al. investigated the role of the Leu56Met variant in the RET gene by clinical follow up of a Danish RET Leu56Met cohort. Using predefined criteria none of the patients exhibited evidence of MEN 2. With an allele frequency of 0.59% the Leu56Met variant suggests that it is a common variant in the population with no pathogenetic importance.

Less frequent are genetic causes of non-medullary thyroid carcinomas (NMTC). About 3% of NFTC are estimated to be of hereditary origin. Most of these tumors arise in the context of syndromic diseases such as familiary adenomous polyposis coli and *PTEN*-Hamartoma-tumor-Syndrome such as Cowden-syndrome. Further entities include Carney-complex, Werner-Syndrome and DICER-1-syndrome. Several single genes are involved or suspected to be to be involved in the pathogenesis of NMTC.

Many studies in recent years gave insight in the important role of genetic alterations in the development and growth progression in papillary thyroid carcinoma (PTC).

Genetic alterations often involve the mitogen-activated protein kinase (MAPK). BRAF as part of the RAF/MAPK pathway is an effector of cell proliferation. Many BRAF mutations have been described in papillary thyroid carcinomas. The BRAF^V600E^ mutation is the most frequent mutation found in papillary thyroid carcinoma and associated with a poorer prognosis, lymph node involvement, extrathyroidal tumor expansion and distant metastases. Due to its high kinase activity secondary genetic alterations such as mutations in phosphoinositide 3-kinase-Akt serine/threonine kinase (PI3K-AKT) pathway may occur leading to more aggressive tumors and an unfavorable clinical outcome for the patients.

Parvathareddy et al. could confirm that BRAF^V600E^ mutations are the most frequent mutations in papillary microcarcinomas. They could detect BRAF^V600E^ mutations in 45.7% of their cases (84 of 184 patients in a middle east population). Beside BRAF ^V600E^ mutations telomerase reverse transcriptase (TERT) promoter mutations play a relevant role in the aggressiveness of tumor growth in papillary thyroid carcinoma. TERT promoter mutations were detected in the series of Parvathareddy in 8.7% cases and were significantly associated with distant metastases and shorter metastasis-free survival in multivariate analysis.

Gao et al. investigated if dysregulation of miRNA plays a role in PCT wit BRAF mutations. In PTC an upregulation of miR-222-3p has already been demonstrated. The actual study showed that miR-222-3p led to more aggressive clinical manifestations of PTC promoted *via* the snail transcription factor.

With surgery and radioiodine therapy most of the patients with differentiated thyroid carcinoma have a favorable outcome. But in radioiodine refractory carcinomas overall survival and progression-free survival time is limited. Clinical management of patients can be improved if the response to radioactive iodine can be detected early. Liu et al. used expression profiles of mRNAs and miRNAs in addition to clinical data of PTC patients. They could apply a two-RNA model (IPCEF1 and hsa-mir-486-5p) associated with the prognosis of RAI-therapy. Together with their RNA-based risk score calculated on the Cox coefficient of the individual RNA and clinical parameters (age at diagnosis and tumor stage) the authors were able to demonstrate high precision in predicting the RAI response of PTC patients.

Rearranged- during transfection (RET) kinase as a protooncogene can also be involved in tumorigenesis of thyroid carcinoma. Besides its role in hereditary forms of medullary thyroid carcinoma, chromosomal RET rearrangements are found almost only in PTC (1). Ret/PTC1 and Ret/PTC 3 are the most common rearrangements. Both forms are found more frequently in younger patients and RET/PTC3 rearrangement has been linked to external radiation.

The knowledge and understanding of the role of genetic alterations in thyroid carcinoma has led to advances in treatment options for patients with radio refractory thyroid carcinomas. Multikinase inhibitors target different pathways including antiangiogenic activity, RET and BRAF^V600E^ and others (2). Lenvatinib and sorafenib have been shown to improve disease-free survival in patients with radio-refractory thyroid carcinoma while cabozantinib has its role as second line therapy in these patients. Selpercatinib is a treatment option for patients with RET fusions in PTC and medullary thyroid carcinomas (3). In MTC, vandetanib and cabozantinib have shown their therapeutic potential already for a couple of years. The most aggressive tumors of the thyroid are anaplastic carcinomas. Understanding the underlying mechanisms of disease and concomitant factors is important for an adequate management of these patients.

Jin et al. used the Surveillance, Epidemiology, and End Results Program (SEER) data base (2004-2015) to compare differences in characteristics between anaplastic thyroid carcinoma (ATC) and primary squamous cell carcinoma of the thyroid (PSCCTh) and establish prognostic models. For patients with PSCCTh (n=124) prognostic factors influencing the cancer-specific survival were age, radiotherapy, multiple primary tumors, and surgery.

For ATC patients (n=1164) these factors were different comprising age, sex, radiotherapy, chemotherapy, surgery, multiple primary tumors, marital status, and distant metastasis. This may lead to the need of different clinical treatment and management in this group of patients.

Conclusively, the understanding of genetic alterations in thyroid carcinoma is growing fast, leading to new treatment options for our patients.

## Author contributions

All authors listed have made a substantial, direct, and intellectual contribution to the work and approved it for publication.

## Conflict of interest

The author declares that the research was conducted in the absence of any commercial or financial relationships that could be construed as a potential conflict of interest.

## Publisher’s note

All claims expressed in this article are solely those of the authors and do not necessarily represent those of their affiliated organizations, or those of the publisher, the editors and the reviewers. Any product that may be evaluated in this article, or claim that may be made by its manufacturer, is not guaranteed or endorsed by the publisher.
